# Network analysis of nitrate-sensitive oral microbiome reveals interactions with cognitive function and cardiovascular health across dietary interventions

**DOI:** 10.1016/j.redox.2021.101933

**Published:** 2021-03-05

**Authors:** Anni Vanhatalo, Joanna E. L'Heureux, James Kelly, Jamie R. Blackwell, Lee J. Wylie, Jonathan Fulford, Paul G. Winyard, David W. Williams, Mark van der Giezen, Andrew M. Jones

**Affiliations:** a College of Life and Environmental Sciences, University of Exeter, UK; b NIHR Exeter Clinical Research Facility, University of Exeter, UK; c College of Medicine and Health, University of Exeter, UK; d School of Dentistry, Cardiff University, UK; e Department of Chemistry, Bioscience and Environmental Engineering, University of Stavanger, Norway

**Keywords:** Oral microbiome, Nitric oxide, Aging

## Abstract

Many oral bacteria reduce inorganic nitrate, a natural part of a vegetable-rich diet, into nitrite that acts as a precursor to nitric oxide, a regulator of vascular tone and neurotransmission. Aging is hallmarked by reduced nitric oxide production with associated detriments to cardiovascular and cognitive function. This study applied a systems-level bacterial co-occurrence network analysis across 10-day dietary nitrate and placebo interventions to test the stability of relationships between physiological and cognitive traits and clusters of co-occurring oral bacteria in older people. Relative abundances of Proteobacteria increased, while Bacteroidetes, Firmicutes and Fusobacteria decreased after nitrate supplementation. Two distinct microbiome modules of co-occurring bacteria, that were sensitive to nitrate supplementation, showed stable relationships with cardiovascular (*Rothia-Streptococcus)* and cognitive (*Neisseria-Haemophilus)* indices of health across both dietary conditions. A microbiome module (*Prevotella-Veillonella*) that has been associated with pro-inflammatory metabolism was diminished after nitrate supplementation, including a decrease in relative abundance of pathogenic *Clostridium difficile*. These nitrate-sensitive oral microbiome modules are proposed as potential pre- and probiotic targets to ameliorate age-induced impairments in cardiovascular and cognitive health.

## 1 Introduction

The human alimentary canal hosts a major microbial ecosystem and the metabolic outputs of these microbes make a significant contribution to host physiology. The symbiotic role of the gut microbiota as a modulator of physiological and cognitive health is well-established [1,2] and cross-sectional studies indicate that a perturbed oral microbial community and poor dental health are associated with impaired cardiovascular and metabolic health [3]. However, relatively little is known about the mechanisms that underlie the host-oral microbiota axis as a potential modulator of human health. One mechanism by which the oral microbiota may influence human health is its contribution to the production of the signalling molecule nitric oxide (^•^NO), which regulates vascular tone, mitochondrial respiration and neurotransmission among many other functions [[4], [5], [6], [7]].

Some facultative and obligate anaerobic oral bacteria reduce nitrate to bioactive nitrite, a key precursor to ^•^NO [8]. Vegetable-rich diets, such as the Mediterranean and ‘Dietary Approaches to Stop Hypertension’ (DASH) diet, are abundant in dietary nitrate and oral bacteria play a key role in the pathway of ^•^NO production from such dietary nitrate. This is particularly important in older age, where the capacity for endogenous ^•^NO production via the canonical enzymic ^•^NO synthase pathway has declined [9,10]. Low ^•^NO availability contributes to the development of arterial hypertension, reduced physical and cognitive functional capacity, and increased morbidity [11,12]. The important role of oral bacterial nitrate reduction as a parallel pathway for endogenous ^•^NO production is demonstrated by studies showing that use of bactericidal mouthwash acutely reduces systemic ^•^NO bioavailability and elevates blood pressure [13,14], and that chronic, frequent use of mouthwash is associated with elevated risk for type II diabetes [15]. Conversely, increased consumption of dietary inorganic nitrate elevates ^•^NO bioavailability and reduces blood pressure [[16], [17], [18]], improves skeletal muscle contractility [19,20] and exercise efficiency [21,22], and enhances brain perfusion and cognitive function [23,24]. Elevation of ^•^NO bioavailability via dietary means, therefore, potentially represents a powerful therapeutic to attenuate cognitive and cardiovascular decline associated with aging [25].

Inorganic nitrate is emerging as an effective intervention to alter the oral microbiome. As a prebiotic dietary intervention, nitrate appears to increase the abundances of bacteria belonging to *Neisseria* and *Rothia* genera [[26], [27], [28]] and decreases abundances of *Prevotella* and *Veillonella* species [27]. Correlational analyses with physiological traits, however, have been limited to single-taxon comparisons [27,29] or aggregate abundances of selected nitrate reducing bacteria [30,31]. Such approaches cannot detect the nuances of complex synergistic and antagonistic relationships that exist among the bacterial ecosystem. In *in vivo* oral ecosystems taxa that contain nitrate-reducing species can be co-exclusive (*e.g. Neisseria* and *Actinomyces;* [32]), and whether the metabolic activity of a given species is beneficial or detrimental to host physiology is determined by the surrounding microbial ecosystem. Therefore, to truly understand how the *in vivo* oral microbial ecosystem might modulate human health, a systems-level microbial co-occurrence network approach is required, in combination with an ‘interventionist’ model that tests the stability of microbiome-trait relationships across two distinct dietary conditions [33,34].

Here, we apply weighted gene co-occurrence network analysis (WGCNA) that was originally developed to explore human genome interactions [35], in a novel context of the oral microbiome interactions with physiological and cognitive traits. The use of WGCNA has been successfully expanded to assess interactions between clinical traits and co-occurring gastric microbiome clusters [36], to compare intestinal microbiomes between healthy and clinical patient cohorts [37], and to assess bacterial interactions within an oral plaque [38]. In this study we systematically reduce the microbiome network data to interpret relationships between co-occurring modules of oral bacteria and the physiological and cognitive traits that are regulated by ^•^NO in older adults. Our application of the WGCNA concept in the form of ‘weighted microbiome co-occurrence network’ considers the oral microbiota as an ecosystem characterized by synergistic and antagonistic relationships among bacteria when exposed to a high-nitrate diet [35,39]. We assessed oral microbiomes by sequencing 16S rRNA genes in saliva samples from healthy older people (70-80 years old) who underwent dietary nitrate and placebo interventions, in conjunction with comprehensive assessments of physiological and cognitive function, as well as muscle and brain metabolism.

The purpose of this study was to determine 1) which co-occurring modules of bacteria in the salivary microbiome of healthy older people were sensitive to dietary nitrate intake, 2) which co-occurring oral microbiome modules correlated with cognitive and physiological traits across dietary interventions with placebo and nitrate, and, 3) which nitrate-sensitive modules represent key biomarkers, and thus probiotic targets, for enhancing cardiovascular and cognitive health. We established eight distinct modules of co-occurring bacteria and revealed profound alterations in relative abundances of specific members of these clusters following dietary nitrate supplementation. We identified several significant correlations between oral bacteria modules and indices of cognitive and physiological function, which represent promising avenues for translational research into biomarkers and therapies for cognitive and cardiovascular impairment in older age.

## 2 Results

### 2.1 Comparison of oral microbiomes between dietary nitrate and placebo conditions

**Fig. 1:**
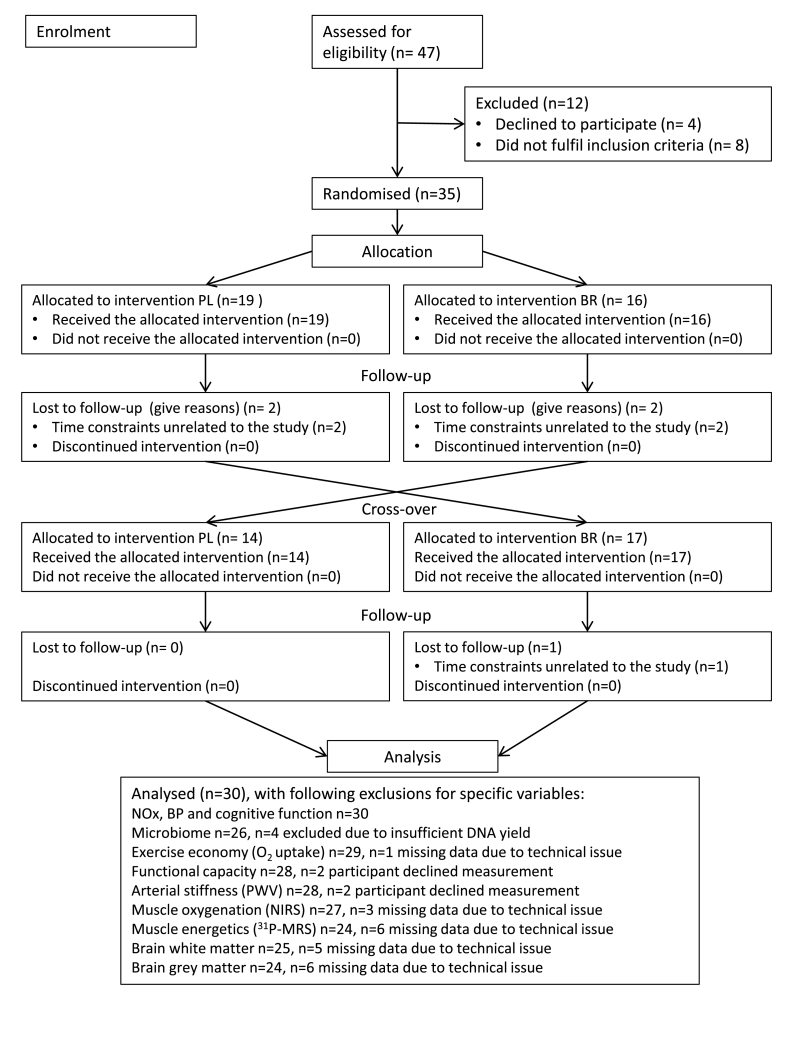
Flowchart illustrating the trial stages from participant enrolment to data analysis. PL, placebo; BR, nitrate.

We surveyed the oral bacterial communities in saliva samples of 30 older people following 10 days of dietary inorganic nitrate (nitrate-rich beetroot juice, ~750 mg NO_3_^−^/d) and placebo (nitrate-depleted beetroot juice, ~1 mg NO_3_^−^/d) supplementation in a randomised, placebo-controlled cross-over design (Fig. 1). Baseline participant characteristics are shown in Table 1. Addenbrooke's Cognitive Examination (ACE-III), that has been validated for the detection of cognitive impairment [40], was used to assess participants' cognitive status at baseline. ACE-III revealed that nine participants had a score of less than 90/100 (range 82–89) indicative of mild cognitive decline. Two participants had a mean arterial pressure (MAP) of >105 mmHg indicative of hypertension. Amplicons of the hypervariable V1–V3 regions of bacterial 16S rRNA gene were amplified and sequenced by synthesis [41], and relative abundances of species-level taxonomic units were assigned using a 97% gene similarity cut-off for pairwise-identity comparisons. Rare operational taxonomic units (OTUs) of less than 10 counts of each OTU in each sample for both placebo and nitrate condition were removed, as well as OTUs which had <5 counts per person in 50% of samples. Thus, in total four samples were excluded, such that the final data set included microbiome data for placebo and nitrate conditions for 26 subjects (Fig. 1).

**Table 1:** Participant characteristics, relative daily NO_3_^−^ dose and salivary flow rate questionnaire (SFR-Q) results (n = 30; men = 13, female = 17).

	Range (mean ± SD)
Age, y	70 - 80 (73 ± 3)
Height, cm	152 - 191 (167 ± 11)
Body mass, kg	51.4 – 103.0 (70.5 ± 13.6)
BMI, kg/m^2^	19.7- 33.1 (25.0 ± 3.3)
ACE-III, total score (/100)	82 - 99 (92 ± 5)
SFR-Q mean score	1.0 - 2.7 (1.6 ± 0.5)
NO_3_^−^ dose (mmol/kgBM/d)	0.12 – 0.24 (0.18 ± 0.03)

BMI, body mass index; ACE-III, Addenbrooke's Cognitive Examination Questionnaire, score out of 100 presented; BMI, body mass index.

**Fig. 2:**
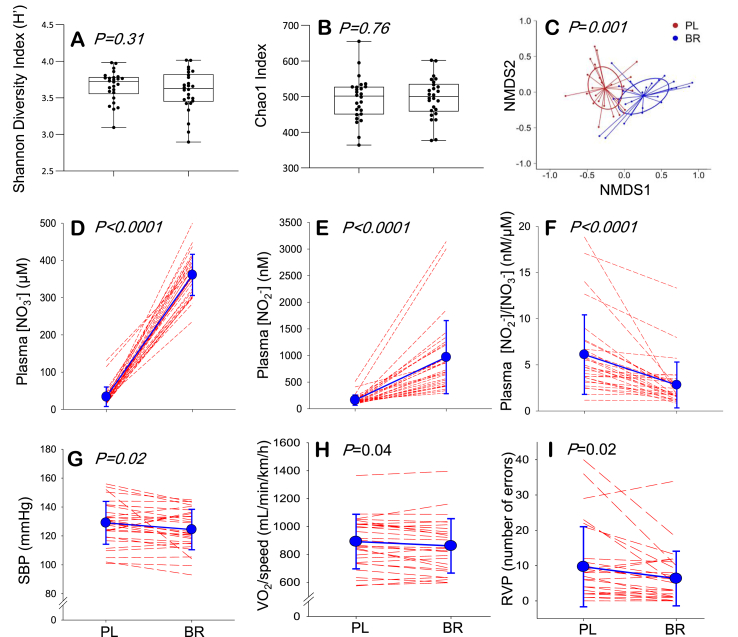
The Shannon diversity index (**A**) and Chao 1 (**B**) indicated no differences in species diversity between placebo (PL) and nitrate (BR) conditions. Non-metric multidimensional scaling (NMDS) revealed that the overall salivary microbiome composition differed between PL and BR (**C**). Significant differences in physiological and cognitive traits between PL and BR included increases in plasma nitrate ([NO_3_^−^]) and nitrite ([NO_2_^−^]) concentration (**D**, **E**); decrease in plasma [NO_2_^−^]/[NO_3_^−^] ratio (**F**); and decreases in systolic blood pressure (SBP) (**G**), pulmonary O_2_ uptake relative to walking speed (**H**), and number of errors in the RVP test of sustained attention (**I**). In panels **D-I** blue symbols and lines indicate mean ± SD and red dashed lines show individual responses. (For interpretation of the references to colour in this figure legend, the reader is referred to the Web version of this article.)

The species richness (number of OTUs/sample), the Shannon Diversity and the Chao-1 index did not differ between placebo and nitrate conditions (Fig. 2 AB). The salivary microbiome compositions were compared between conditions using non-metric multidimensional scaling (NMDS) ordination based on Bray-Curtis dissimilarity, coupled with t-tests and Benjamini-Hochberg false discovery rate correction [42]. This analysis revealed a shift in microbial community between conditions, as indicated by distinct clustering of placebo and nitrate samples (Fig. 2 C). Significant phylum-level shifts in relative abundance following nitrate supplementation included a marked increase in Proteobacteria, which occurred in concert with decreases in Firmicutes, Bacteroidetes and Fusobacteria. Overall, relative abundances of 23 genera and 24 species were different between placebo and nitrate conditions following α-adjustment for multiple comparisons (Table S1). Nitrate supplementation increased the abundances of 10 species, while decreasing the abundances of 14 others.

### 2.2 Comparison of ^•^NO bioavailability and physiological and cognitive function between dietary nitrate and placebo conditions

Differences between placebo and nitrate conditions in variables that reflect ^•^NO bioavailability and cognitive and physiological function were assessed using paired-samples t-tests (Table S2). Plasma concentrations of ^•^NO biomarkers, nitrate and nitrite, were elevated and systolic blood pressure (SBP) was reduced after nitrate supplementation (Fig. 2 D, E). The plasma [nitrite]/[nitrate] ratio was lower in nitrate compared to placebo condition, which may reflect decreased efficiency of nitrate reduction, nitrate-reductase saturation, and/or alterations in nitrite oxidation and reduction pathways present in other tissues that contribute to plasma nitrate and nitrite concentrations (Fig. 2 F). The O_2_ cost of sub-maximal treadmill walking was lower after nitrate supplementation than placebo, indicating enhanced exercise efficiency (*i.e.*, lower O_2_ cost for a given external work rate). There was no difference between conditions in maximal rate of skeletal muscle mitochondrial ATP resynthesis ($\dot{Q}$max) which was estimated in the quadriceps muscles using quantitative ^31^Phosphorous magnetic resonance spectroscopy (^31^P-MRS). Physical functional capacity, assessed via a sit-to-stand test and a 6-min walk test (6MWT), was not altered by the interventions (Table S2). Cognitive performance in Rapid Visual Information Processing (RVP) test, a measure of sustained attention, was enhanced following nitrate supplementation compared to placebo (Fig. 2 I). There were no differences in cognitive performance tests of short-term memory (number recall and serial subtractions), selective attention (Stroop test) and information processing speed (choice-reaction time). ^1^H-MRS brain scans revealed no differences between dietary conditions in *N*-acetyl aspartate (NAA), choline (Ch), creatine (Cr) or myo-inositol (mI) in occipito-parietal grey matter or in left frontal white matter (Table S2).

### 2.3 Weighted oral microbiome co-occurrence network analysis and its relationship with cardiovascular and cognitive traits

To identify biologically relevant correlations between the oral microbiome and indices of cardiovascular and cognitive health that were stable across placebo and nitrate conditions, we adapted WGCNA to create a weighted microbiome co-occurrence network [35]. Using a signed network where modules represented positively correlated OTUs and a soft thresholding value of >0.8, a total of eight distinct microbiome modules (MM1-MM8) were identified and 26 OTUs were unassigned to any module (Fig. S1). The size of the modules ranged from 24 to 243 taxonomic units, and six of the modules included OTUs that were sensitive to dietary nitrate (Table 2). Most of the OTUs that increased with nitrate supplementation clustered together in MM5, with the exception of *Rothia mucilaginosa* (MM6) and *Phycisphaera mikurensis* (MM1), and two OTUs that were unassigned to a network (*i.e.*, did not form significant correlations with neighbouring OTUs; Table 2). In contrast, most OTUs that decreased after nitrate supplementation clustered together in MM2, with the few other declining OTUs scattered in MM1, MM3 and MM4.

**Table 2:** The distribution of nitrate-sensitive bacteria among microbiome modules 1–8 (MM1-MM8) and module-driver species exceeding LDA score threshold of 2. Bold font indicates nitrate-sensitive module drivers. * No taxonomic unit exceeded LDA threshold of 2. PL, placebo; BR, nitrate.

Microbiome module	Number of taxonomic units	Nitrate-sensitive species (% change from PL to BR)	Module-driver species
**MM1** Turquoise	243	*Bacillus amyloliquefaciens* (−37%) *Phycisphaera mikurensis* (+118%) *Ruminococcus bromii* (−56%) ***Spirochaeta africana* (-66%)**	*Clostridium botulinum* *Selemonas ruminantium* ***Spirochaeta africana***
**MM2** Green	48	*Atopobium parvulum* (−60%) ***Clostridium difficile* (-79%)** ***Megasphaera elsdenii* (-80%)** ***Prevotella intermedia* (-46%)** *Prevotella melaninogenica* (−56%) ***Ruminococcus torques* (-78%)** *Veillonella parvula* (−63%)	***Clostridium difficile*** ***Megasphaera elsdenii*** ***Prevotella intermedia*** ***Ruminococcus torques***
**MM3** Pink	24	*Tetragenococcus halophilus* (−30%)	*
**MM4** Blue	71	*Butyrivibrio proteoclasticus* (−41%) *Clostridium phytofermentans* (−47%) ***Roseburia hominis* (-52%)**	*Bacteroides vulgatus* *Campylobacter curvus* ***Roseburia hominis*** *Tannerella forsythia*
**MM5** Yellow	57	*Capnocytophaga orchracea* (+96%) *Flavobacterium indicum* (+183%) *Neisseria lactamica* (+175%) *Neisseria meningitidis* (+305%) *Nitrosococcus halophilus* (+115%) *Ornithobacterium rhinotracheale* (+109%)	*Haemophilus parainfluenzae* *Neisseria gonorrhoeae* ***Neisseria lactamica*** *Nitrosococcus halophilus* *Riemerella anatipestifer*
**MM6** Brown	71	*Rothia mucilaginosa* (+259%)	***Rothia mucilaginosa*** *Streptococcus pyogenes*
**MM7** Red	46	–	*
**MM8** Black	24	–	*
Unassigned Grey	26	*Leptothrix cholodnii* (+628%) *Variovax paradoxus* (+544%)	

**Fig. 3:**
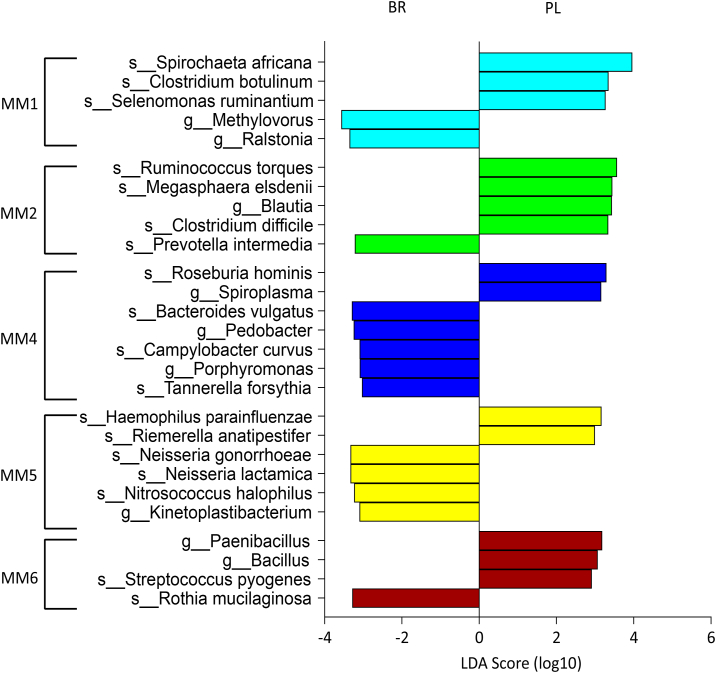
Linear discriminant analysis (LDA) effect size (LEfSe) was used to identify operational taxonomic units (OTU) driving the differences between placebo (PL) and nitrate (BR) conditions within oral microbiome modules 1–6 (MM1-MM6). Within MM3, MM7 and MM8 no OTU exceeded the logarithmic LDA score threshold of 2.

To identify module-driver OTUs that showed the highest connectivity to other OTUs within a given module we used linear discriminant analysis (LDA) effect size (LEfSe), a method that has been validated for biomarker identification within the human microbiome [43]. The module-driver OTUs for each module exceeding a logarithmic LDA score threshold of 2 are illustrated in Fig. 3. We found that eight module-driver OTUs were nitrate-sensitive, including two OTUs that increased (MM5: *Neisseria lactamica*; MM6: *R. mucilaginosa*) and six OTUs that decreased (MM1: *Spirochaeta africana*; MM2: *Clostridium difficile*, *Megasphaera elsdenii*, *Prevotella intermedia*, *Ruminococcus torques*; MM4: *Roseburia hominis*) with dietary nitrate supplementation.

**Fig. 4:**
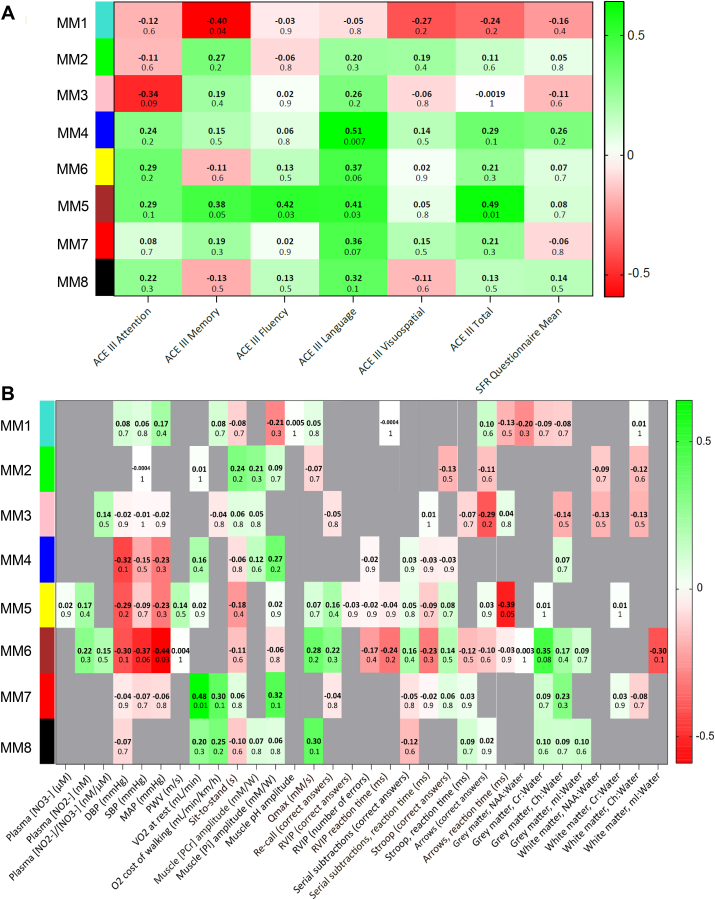
**A**: Eigengene correlation network between the Addenbrooke's Cognitive Examination (ACE-III) cognitive traits at baseline and microbiome modules (MM1-MM8) within the PL condition. **B**: Consensus correlation network between oral microbiome modules and 32 cognitive and physiological traits. Only three significant consensus module-trait relationships persisted across placebo and nitrate conditions: MM5 correlated with reaction time in the ‘information processing’ cognitive function test, MM6 correlated with MAP, and MM7 correlated with pulmonary O_2_ uptake at rest. The colour scale on the right-hand side of each panel indicates the strength of positive (green) and inverse (red) correlations. (For interpretation of the references to colour in this figure legend, the reader is referred to the Web version of this article.)

To screen for functional significance of the microbiome modules, we summarised each module according to an intramodular hub gene (‘eigengene’, *i.e.*, the first principal component of module microbiome expression) and created eigengene correlation networks with physiological and cognitive traits. The ACE-III characterisation was performed at baseline only, since it was not expected that cognitive status would change during a 10-day dietary intervention. ACE-III cognitive traits were therefore analysed as part of the PL condition eigengene network. MM6 positively correlated with ACE-III total (*r* = 0.45, *P* = 0.01), fluency (*r* = 0.42, *P* = 0.03) and language scores (*r* = 0.41, *P* = 0.03), MM4 positively correlated with ACE-III language score (*r* = 0.51, *P* = 0.007) and MM1 inversely correlated with ACE-III memory score (*r* = −0.40, *P* = 0.04) (Fig. 4A). ACE-III total score correlated with physical functional capacity (6MWT, *r* = 0.49, *P* = 0.017), MAP (*r* = −0.47, *P* = 0.0090), BMI (*r* = −0.46, *P* = 0.011), memory and concentration (serial subtractions, *r* = 0.46, *P* = 0.011), and brain white matter Ch (*r* = 0.42, *P* = 0.035). Compared with the participants with an ACE-III total score of >90 (n = 21), participants with mild cognitive impairment (n = 9) had a higher body mass index (BMI) (27 ± 3 vs 24 ± 3 kg/m^2^) and SBP (137 ± 10 vs 126 ± 15 mmHg), and lower brain white matter Ch:Water ratio (0.62 ± 0.21 vs 0.92 ± 0.34) (all *P* < 0.05).

Next, we surveyed 520 module-trait eigengene correlations (8 modules x 32 traits x 2 dietary conditions) and found 19 significant relationships within the placebo and 19 within the nitrate condition (Fig. S2 AB). However, most of these significant correlations were not stable across both dietary conditions, suggesting that they may be spurious rather than causal. To assess the stability of the microbiome module-trait correlations across dietary conditions we further reduced the data to a consensus network [35] and found that only three significant correlations remained: MM5 correlated with reaction time in the ‘information processing’ cognitive function test (*r* = −0.39, *P* = 0.05), MM6 correlated with MAP, (*r* = −0.44, *P* = 0.03), and MM7 correlated with $\dot{V}$O_2_ at rest (*r* = 0.48, *P* = 0.01) (Fig. 4B). Plasma ^•^NO biomarkers, muscle bioenergetic indices, and brain metabolites showed several significant correlations with microbiome modules in either the placebo or nitrate condition (Fig. S2), but none of these evidenced stability in the consensus network (Fig. 4B).

## 3 Discussion

Microbiome-diet interactions are emerging as a therapeutic target for the maintenance of cardiovascular and cognitive health in older age [44,45]. While there is robust evidence to suggest that the oral microbiota as a whole plays a key role in the maintenance of systemic ^•^NO homeostasis, attempts to link individual bacterial species to ^•^NO-mediated functional outcomes miss the context of complex synergistic and antagonistic interactions within the oral bacterial community. Therefore, causal inferences drawn from cross-sectional comparisons have limited translational potential for advancing healthy ageing in humans.

We applied a systems biology approach to detect biologically-relevant features of the nitrate-sensitive oral microbiome in 70–80 year-old men and women. Our network-driven analysis systematically reduced the bacterial 16S rRNA sequencing data to reveal two modules of co-occurring bacteria (MM5 and MM6) that exhibited both 1) nitrate-sensitivity and 2) stable correlations with blood pressure (MM6) or cognitive function (MM5) across two distinct dietary conditions. In addition, we identified another microbiome module (MM7) that was not nitrate-sensitive, but showed a stable correlation to O_2_ uptake at rest.

There is a remarkable consistency in the clustering of oral microbiota irrespective of age and ethnicity of the subject population as well as the sequencing and bioinformatics approaches taken to analyse microbiome data (46, 47, 48; present study). The number of oral microbiome modules identified has varied across studies (~2–8), but the most consistently identified modules include the *Prevotella-Veillonella* (MM2; 46, 47, 48), *Neisseria-Haemophilus* (MM5; 47, 48), and *Streptococcus-Rothia* clusters (MM6; 47, 48). We showed that it was possible to manipulate the relative proportions of these ‘universal’ oral microbiome clusters via a simple, short-term dietary nitrate supplementation in a population of 70–80 year-old individuals who followed their habitual omnivorous diet throughout the study; specifically, the *Prevotella-Veillonella* cluster was diminished, while the *Neisseria-Haemophilus* and *Streptococcus-Rothia* clusters thrived under nitrate supplementation. In terms of oral health, the *Prevotella-Veillonella* module has been associated with proteolytic metabolism and an early inflammatory state with a dysbiotic prognosis [48]. This module is also predominant in smokers [49], and positively associated with periodontitis [50] and pneumonia in older people [47]. In contrast, a *Neisseria-Haemophilus* dominated oral microbiome has been associated with periodontal health, younger age, lower BMI, and abstinence from smoking [49]. *Neisseria*, *Haemophilus* and *Rothia* are among bacteria that dominate the oral microbiomes of vegans, while the omnivorous diet favours *Prevotella* and *Streptococcus* [46]. Our findings from a placebo-controlled, cross-over dietary intervention with nitrate-rich and nitrate-depleted beetroot juice indicate that the greater *Neisseria/Prevotella* ratio reported in vegans compared to omnivores [46] was likely due to differences in dietary nitrate intake. Collectively, this ecosystemic shift following dietary nitrate supplementation away from *Prevotella-Veillonella* and towards dominance by *Neisseria-Haemophilus* and *Streptococcus-Rothia* modules, may have positive implications for oral and systemic health.

Alongside the shift in the oral microbiome, the dietary nitrate intervention resulted in beneficial changes in systemic ^•^NO bioavailability, blood pressure, O_2_ cost of walking, and sustained attention that were indicative of improved cardiovascular, metabolic and cognitive function. A key strength of the current study design was the ‘interventionist’ approach [34] that enabled us to test the stability of correlations between co-occurring oral microbiome modules and physiological and cognitive traits across two distinct dietary conditions using a consensus network [35]. We found that the inverse correlation between MM6 (*Streptococcus-Rothia* module) and MAP, was sufficiently stable to persist when subjected to the consensus network. This module was mainly made up of Firmicutes and Actinobacteria, including 24 *Streptococcus* species (*e.g*. *S. mitis, S. parasanguinis* and *S. salivarius*) and two *Rothia* species (*R. dentocariosa* and *R. mucilaginosa*), but among the module drivers within MM6, only *R. mucilaginosa* was sensitive to nitrate supplementation (Table 2). The presence of streptococci, a core component in a healthy oral ecosystem, correlates positively with markers of vascular health (high-density lipoprotein and apolipoprotein AI; 51) and *R. mucilaginosa* is a potent nitrate-reducer [8], that positively correlates with systemic ^•^NO biomarkers [27]. The sensitivity of MM6 to dietary nitrate is of considerable therapeutic significance, given that elevated blood pressure represents a primary risk factor for cardiovascular diseases, the leading cause of mortality in the developed world [52]. A diet rich in inorganic nitrate throughout the lifespan and particularly in older age, may have a beneficial influence on the oral microbiota with associated reduction in the risk for developing cardiovascular diseases.

There was a positive consensus correlation between O_2_ uptake at rest and MM7, a relatively small bacterial module that was not nitrate-sensitive and had no species-level module drivers exceeding the LDA score threshold. The metabolic underpinnings of this stable correlation across the two dietary conditions are unclear. The O_2_ uptake at rest, measured with the participant standing astride the treadmill prior to the commencement of exercise test, reflects the body's metabolic rate, which is influenced by lean tissue mass, metabolic properties of tissue, and environmental conditions. Although nitrate supplementation resulted in a considerable increase in plasma ^•^NO biomarkers, there were no significant consensus-level correlations between nitrate or nitrite concentration and any microbiome module. This result may reflect the fact that ^•^NO is omnipresent in the human body and is not produced solely through the nitrate-nitrite-NO pathway involving the oral microbiota, but also via the NOS-enzyme pathway and the nitrate-reductase activity of the gut microbiota [53]. It should also be noted that total bacterial mass and metabolic activity, which are not quantified by 16S sequencing, also influence the volume of oral nitrite production and should be considered alongside relative abundances of bacteria.

The first signs of cognitive decline predominantly manifest as deficits in executive function, sustained attention, and information processing speed [54]. Our findings that a short-term dietary nitrate intervention improved sustained attention (reduced number of errors in the RVP test), and that information processing speed (in Decision-Reaction test) was inversely correlated with the nitrate-sensitive *Neisseria-Haemophilus* module (MM5), therefore have potential for the development of interventions to delay the progression of cognitive decline. It was also noteworthy, that the baseline cognitive status assessed through ACE-III questionnaire correlated with the *Streptococcus-Rothia* module (MM6), and that *P. intermedia,* a module driver (MM2) that has been implicated as a predictor of cognitive decline [55], decreased significantly after nitrate supplementation. The known periodontal pathogens *Porphyromonas gingivalis*, *Treponema denticola* and *Tannerella forsythia* that have emerged as correlates of cognitive decline and Alzheimer's disease [56] clustered together in MM4, which diminished after nitrate supplementation. Low relative abundances of these pathogens (~0.02–0.06%) are representative of the comparatively good health status of the study cohort. Further randomised controlled trials are warranted to test the robustness of the cognitive state versus *Streptococcus-Rothia* module relationship in an interventionist consensus network, and to explore the use of dietary nitrate as a preventative long-term intervention for attenuating progression of cognitive decline.

Elevated blood pressure is a risk factor for cognitive decline in middle-aged and older populations up to approximately 84 years of age [57]. Accordingly, both MAP and cognitive status at baseline correlated with the *Streptococcus-Rothia* module. A common denominator in cognitive and cardiovascular decline in older age is a chronic low-level inflammation [58]. Oral bacteria might underpin inflammation by affecting host metabolism (including ^•^NO homeostasis) while residing in the oral cavity and/or via bacterial migration beyond their native habitat, *e.g.*, to the vascular endothelium forming atherosclerotic plaques [51]. Atherosclerosis risk is linked to the oral microbiome [59], and a meta-analysis of 63 studies and 1791 patients identified *P. intermedia* among five oral bacteria found uniquely in coronary atherosclerotic plaque [60]. Decreased ^•^NO bioavailability encourages leukocyte recruitment and systemic inflammation, and indeed, dietary nitrate supplementation that elevates ^•^NO bioavailability has been shown to attenuate inflammation and result in a more stable plaque phenotype in a mouse model of atherosclerosis [61]. It is also interesting to note that the dietary nitrate supplementation decreased four microbiome module drivers including *P. Intermedia* and *Clostridium difficile* in MM2, which has been characterized by proteolytic and pro-inflammatory metabolism [48] and which contains bacteria that might stimulate atherosclerotic processes in the vasculature when the oral-blood barrier is breached. The novel observation that dietary nitrate supplementation decreased the relative abundance of oral *C. difficile,* possibly arising from cidal activity of reactive oxides produced from acidified nitrite [62], may have implications, for example, for infection prevention during proton pump inhibitor treatment in clinical practice and in long-term-care facilities [63].

Sequencing of the ribosomal 16S genes provides a cost-effective characterisation of the microbiome and enables useful comparisons to be made between populations or conditions. However, 16S sequencing has modest accuracy in identification of species level OTUs, and unlike whole genome sequencing (WGS), it does not enable verification of nitrate-reductase genes in given OTUs. Therefore, while our results provide an instructive overview of the nitrate-sensitive host-oral microbiome network, further research is required to confirm specific species-level biomarkers for targeted probiotics development. It should be noted when drawing comparisons between studies, that the quantities as well as relative abundances of bacteria vary between different oral sites, with nitrate-reducing bacteria being most abundant on the dorsum of the tongue [8]. We chose saliva sampling because it provides an overall characterisation of the microbial communities present across all oral sites, and the relative abundances of dominant bacteria in saliva are closely correlated to those on the tongue dorsum [64]. The supplementation period of 10 days is deemed sufficiently long to yield significant changes in key variables measured in the present study, where we aimed to characterise a broad range of physiological and cognitive responses to nitrate supplementation that may correlate with the oral microbiome. While we excluded participants with known periodontal diseases, the oral health status of participants was self-reported and not based on oral examination. Future research is warranted to explore the influence of various lifestyle factors (*e.g*., diet, oral hygiene routines) on the efficacy of prebiotic nitrate supplementation, as well as the effects of longer supplementation periods on cognitive status using bespoke cognitive test batteries.

The principal novel contribution of this study was the identification of co-occurring modules of bacteria (MM5 and MM6), which exhibited consistent correlations to indices of human health across dietary interventions. Characterisation of the *in vivo* oral ecosystem in this way is a major advance to using isolated OTUs [27,29] or arbitrarily grouped OTUs [30,31] for correlational analyses with physiological variables. Importantly, we also showed that nitrate supplementation diminished MM2, which has been associated with inflammatory metabolism [48] and contained oral pathogens such as *C. difficile*. The network approach originally developed for human genome research applied in a novel context of the oral microbiome to account for complex interactions among oral bacteria, enabled us to identify *Neisseria* (MM5) and *Rothia* (MM6) OTUs, which: 1) both increased significantly with nitrate supplementation, 2) represented module-drivers with the most connectivity to their neighbouring OTUs, and 3) presented stable correlations with cardiovascular or cognitive function. While these OTUs may be considered to have promise as new probiotics, it is important to note that a single-taxon focus can be misleading because the gene expression and signalling cascades of a given bacterial species are modulated by its co-inhabitants in the collective microbial ecosystem [65]. Therefore, for optimal ecosystem-wide effects, potential probiotics should be combined with dietary nitrate supplementation as a prebiotic ancillary, as well as other bacterial species identified as module drivers (irrespective of nitrate-reductase activity) within the desired microbiome module.

## 4 Materials and methods

### 4.1 Participants

This trial (NCT03473678) was approved by the institutional Research Ethics Committee (Sport and Health Sciences, University of Exeter) and conformed to the World Medical Association (WMA) Declaration of Helsinki. Participants gave their informed, written consent after the experimental procedures had been discussed, and potential risks and benefits of participation had been explained. Exclusion criteria included known illnesses, including oral diseases, the wearing of dentures, and tobacco and prescription medication use. Participants were instructed to arrive at the laboratory in a rested and fully hydrated state, at least 3 h postprandial, and to avoid strenuous exercise in the 24 h preceding each laboratory visit. Participants were asked to follow their habitual diet and physical activity pattern and refrain from using mouthwash throughout the study. Participants were also advised to avoid caffeine and alcohol intake 6 and 24 h before each visit. All tests were performed at approximately the same time of day (±2 h) for each subject. Participants completed the Addenbrooke's Cognitive Examination III [40] and salivary flow rate [66] questionnaires at baseline (Table 1) and were familiarised with test protocols for physiological and cognitive traits.

### 4.2 Dietary interventions

Participants were assigned in a double-blind, randomised, crossover design to receive 10 days of dietary supplementation with concentrated NO_3_^−^ -rich BR (2 × 70 ml d^−1^, organic beetroot juice each containing ~595 mg NO_3_^−^, Beet it, James White Drinks, Ipswich, UK) and NO_3_^−^ -depleted PL (2 × 70 ml d^−1^, organic beetroot juice containing ~1 mg NO_3_^−^, Beet it, James White Drinks, Ipswich, UK). The PL beverage was created by passage of the juice, before pasteurisation, through a column containing Purolite A520E ion exchange resin, which selectively removes NO_3_^−^ ions [67]. The PL product was identical to the BR in appearance, taste and smell. Participants were instructed to consume one of the 70 ml beverages in the morning and the other in the afternoon each day, and on laboratory visit days, to consume their final beverage 2.5 h prior to the laboratory visit. A wash-out period of at least 72 h separated each supplementation period and participants were instructed to maintain their habitual daily activities and food intake throughout the study. Participants attended the laboratory on days 8, 9 and 10 of each supplementation period for the assessment of physiological and cognitive traits. Upon arrival at the laboratory on each of these visits, venous blood samples were drawn, resting BP was measured, and a saliva sample collected (by expectoration, without stimulation) for the assessment of the salivary microbiome. Plasma [NO_3_^−^] and [NO_2_^−^] were assessed using ozone-based chemiluminescence [68] and the mean values of three measurements was calculated. The three saliva samples for each individual were pooled for analysis of the salivary microbiome.

### 4.3 Physiological traits

Venous blood samples were drawn from the antecubital vein and seated blood pressure was measured in accordance with European Society of Hypertension guidelines ([69]; Dinamap PRO 100V2, GE Medical Systems Information Technology, Tampa, USA). During a baseline visit, participants completed an incremental exercise test on a treadmill (Woodway ELG 55, Woodway Gmbh, Weil am Rhein, Germany) for the assessment of the gas exchange threshold (GET; 17). During experimental visits, participants were asked to complete a ‘Sit to Stand 10 Test’ (stand from a standard chair 10 times as quickly as possible), and a walking exercise test on a treadmill for the determination of pulmonary oxygen uptake ($\dot{V}$O_2_; Oxycon Pro, Jaeger, Hoechburg, Germany). The protocol involved three 6-min bouts of low-intensity walking at a speed equivalent to 90% of the GET [17]. Each exercise bout involved an abrupt transition to the target speed initiated from a slow walking baseline (1 km h^−1^), with the three exercise bouts separated by 10 min of passive recovery. Following a 15 min passive recovery period, the participants completed a 6-Min Walk Test (6MWT) on a straight, flat track to assess functional capacity in accordance with the American Thoracic Society guidelines [70].

### 4.4 Cognitive traits

Participants were asked to complete five computer-based cognitive function tests which assessed the speed and accuracy of response during cognitively demanding tasks. Participants completed: 1) the *Rapid Visual Information Processing test* (RVP), to assess sustained attention; 2) the *Number Recall test*, to assess short-term working memory; 3) the *Stroop test* to assess information processing speed, executive abilities and selective attention; 4) the *Decision-Reaction test*, to assess information processing speed; and 5) the *Serial Subtractions test* by serial threes and serial sevens, to assess concentration and memory.

### 4.5 ^31^P-MRS of the quadriceps muscle and ^1^H-MRS of the brain

Participants were asked to complete single-leg, knee-extension exercise tests while lying prone in the bore of a 1.5 T superconducting MR scanner (Gyroscan Clinical Intera, Philips, The Netherlands). First, muscle phosphocreatine ([PCr]), inorganic phosphate ([Pi]), adenosine triphosphate ([ATP]) and [pH] dynamics were assessed during two low-intensity knee-extension exercise bouts (each 4 min). Then, participants were asked to complete two 24-s high-intensity knee-extension exercise bouts, separated by 3 min 36 s rest, to assess muscle phosphocreatine (PCr) recovery kinetics, which represents the maximal rate of mitochondrial ATP resynthesis, and thus an index of muscle oxidative capacity ($\dot{Q}$max; [71]). During the same visit to the MR scanner, brain metabolites including *N*-acetyl aspartate (NAA), creatine (Cr), choline (Ch), myo-Inositol (mI) of left frontal white matter and occipito-parietal grey matter were measured using ^1^H MRS following the methods by Kelly et al. [17].

### 4.6 DNA extraction and 16S illumina sequencing

Genomic DNA (gDNA) from the saliva samples was extracted using the method by Goode et al. [41]. Briefly, DNA stabilisation buffer and cell lysis solution was added to the saliva samples, mixed, and incubated at room temperature for 30 min. RNAse solution was added to the samples and these were incubated on ice for the removal of RNA. Proteinase K solution and protein precipitation solution were then added to remove proteins and lipids. For isolation of gDNA, glycogen solution and isopropanol were added and centrifuged for 30 min at 3000 *g* and 4 °C. The gDNA was purified using an ethanol wash and rehydrated with Tris-EDTA. DNA concentration was quantified using Qubit high-sensitivity fluorescence detection (Qubit 3.0, ThermoFisher Scientific, Waltham, MA). Library preparation employed a NEXTflex 16S V1 – V3 Amplicon-Seq Kit (Bioo Scientific, Austin, TX). 16s V1–V3 rDNA was selectively amplified using universal primers. Following AMPure® XP bead cleanup (Becton Dickinson, Franklin Lakes, NJ), a subsequent PCR with indexing primers to identify individual samples, containing Illumina flow cell binding sites, was performed. Paired-end 300 metagenomic next generation sequence analysis was performed on the MiSeq Illumina platform (Illumina, San Diego, CA) using v3 MiSeq reagents. Nucleotide sequence data in FASTQ format was trimmed and processed by the Kraken 2 Taxonomic Sequence Classification System [72]. Variations in the V1–V3 regions enabled taxonomic identification.

### 4.7 Weighted microbiome co-occurrence network analysis

WGCNA was adapted to group highly correlated OTUs and correlate the groups with physiological traits (WGCNA R version 6.3). Data were filtered by removing counts of less than five in >50% of the samples and transformed in accordance with methods described by Langfelder & Horvath [35]. An analysis of the network topology was performed using a signed consensus network for PL and BR where the soft thresholding value was at least 0.8 in PL and BR. In module identification, spurious associations were minimised by transforming the adjacency matrix to a topological overlap matrix. A hierarchical clustering tree was used for OTU clustering and module identification (Fig. S2). Module eigengenes for PL and BR were correlated with the physiological traits. A consensus network was used to determine the concordance and differences between the networks [35]. Linear discriminant analysis (LDA) of effect size (LefSE) was used to identify key biomarkers within in each module and compare the statistically different features in PL and BR. The ‘all-against-all’ strategy for multiclass analysis was used with a logarithmic LDA score threshold of 2 [43].

### 4.8 Statistical analyses

Differences between placebo and nitrate conditions in ^•^NO bioavailability, and physiological and cognitive traits were assess using paired-samples t-tests. Non-metric multidimensional scaling (NMDS) was used to assess the level of similarity in microbiomes between conditions using non-parametric relationships and were analysed using ADONIS (Vegan R Software). Differences between conditions in bacteria that made up >0.01% of total bacteria were assessed using Benjamini-Hochberg-adjusted paired t-tests (R statistical software; 42). The Shannon-Wiener diversity index (H’) was used to explore differences in diversity (Vegan R Software). Statistical significance was defined as *P ≤* 0.05.

## Declaration of competing interest

None.
