# Supplementary material for: Network analysis of nitrate-sensitive oral microbiome reveals interactions with cognitive function and cardiovascular health across dietary interventions
Source: Redox Biol. 2021 Mar 5;41:101933. doi: 10.1016/j.redox.2021.101933 (PMC7970425; doi:10.1016/j.redox.2021.101933)
Supplement: Multimedia component 1 [file mmc1.pdf]

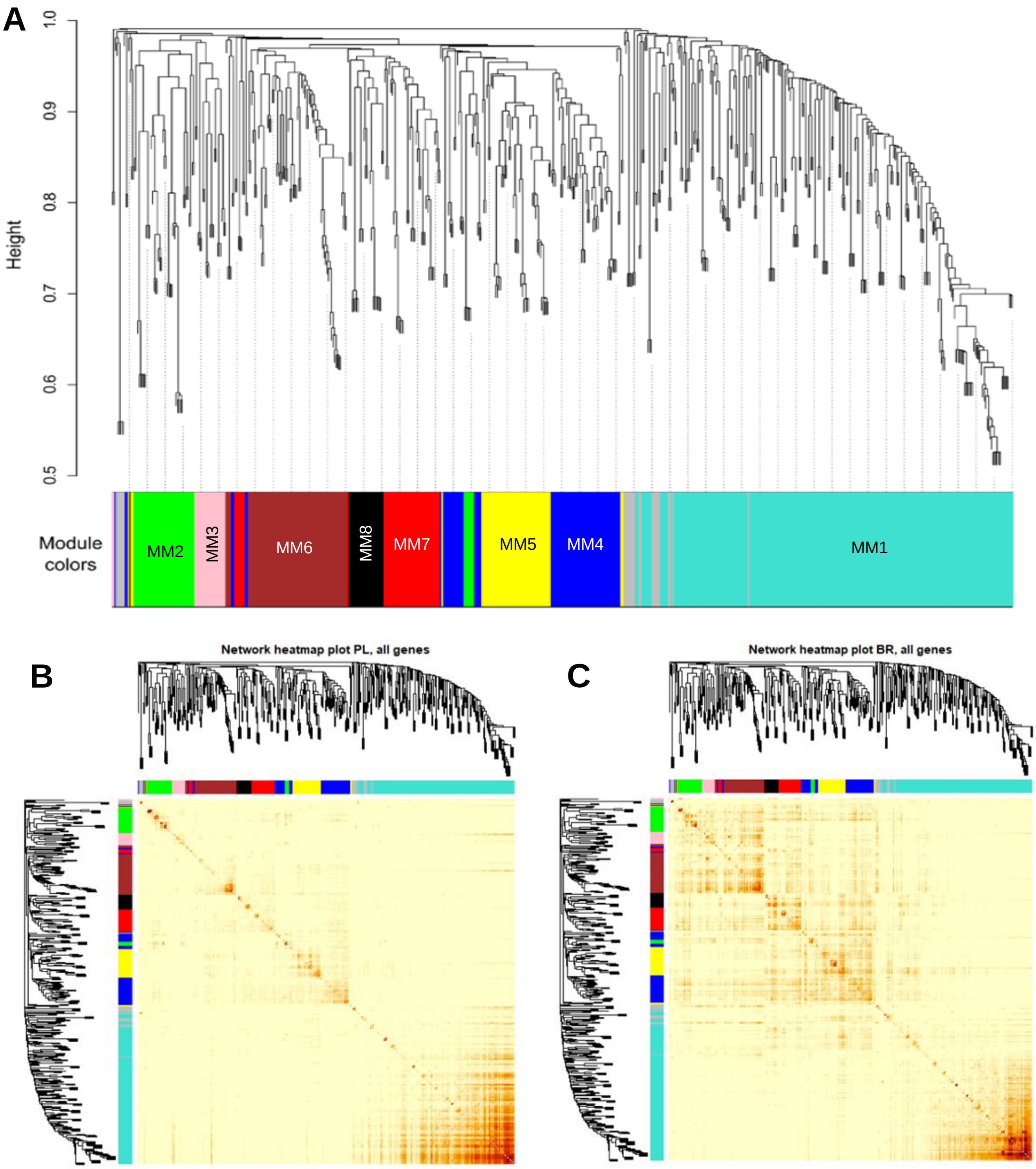

Fig. S1. A: Consensus gene dendrogram. Using a signed network where modules represent positively correlated taxonomic units (power >0.80), a total of eight distinct microbiome modules (MM1-MM8) were identified. **B, C**: Heatmaps illustrating the microbiome networks in placebo (PL) and nitrate conditions (BR). A darker colour indicates a high overlap between the taxonomic units. The horizontal and vertical colour blocks represent the modules MM1-MM8.
