# Supplementary material for: Network analysis of nitrate-sensitive oral microbiome reveals interactions with cognitive function and cardiovascular health across dietary interventions
Source: Redox Biol. 2021 Mar 5;41:101933. doi: 10.1016/j.redox.2021.101933 (PMC7970425; doi:10.1016/j.redox.2021.101933)
Supplement: Multimedia component 2 [file mmc2.pdf]

A

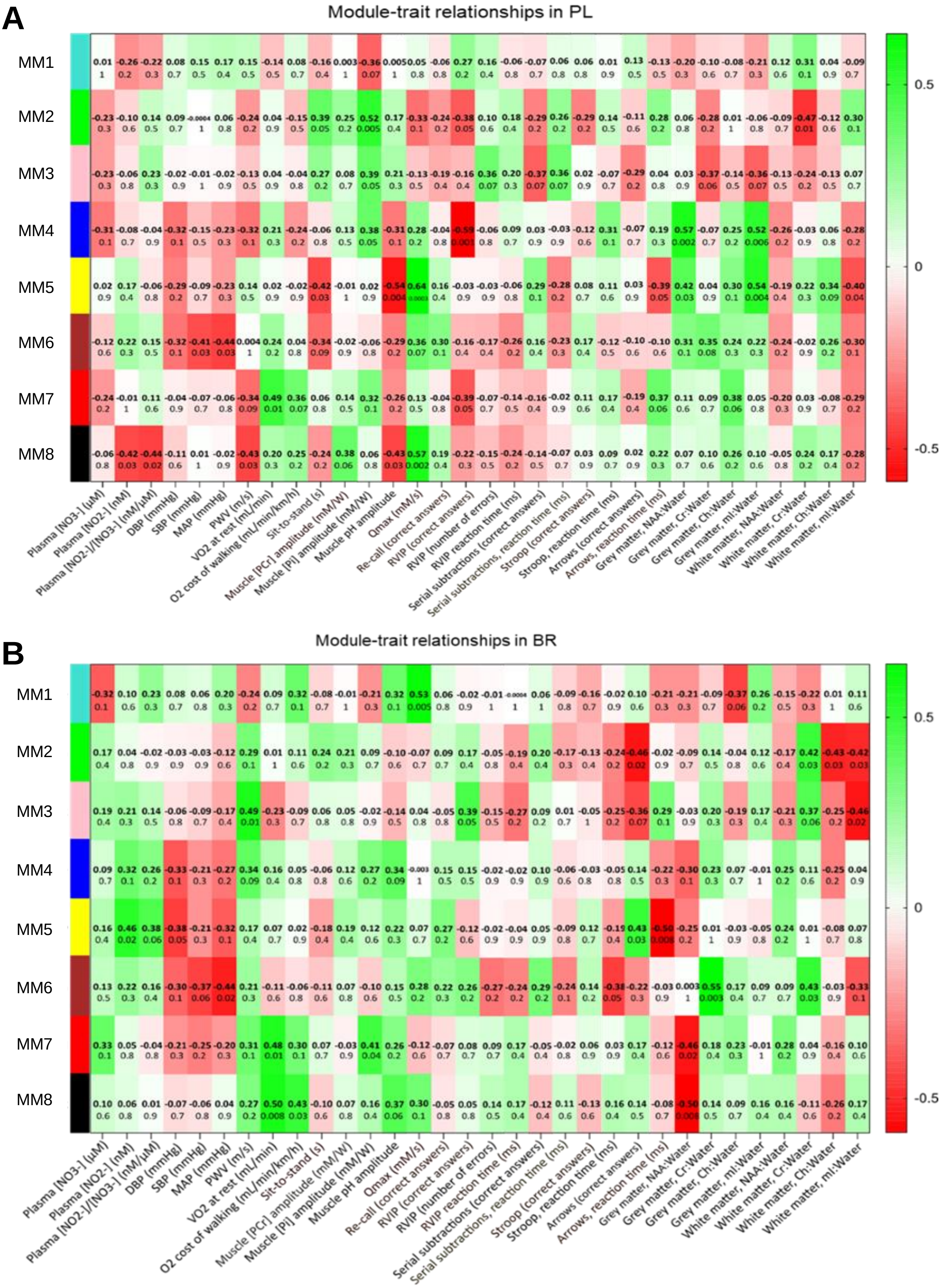

B

Fig. S2. Eigengene correlation networks between oral microbiome modules (MM1-MM8; vertical axis) versus 32 physiological and cognitive traits (horizontal axis) within placebo (PL; A) and nitrate (BR; B) conditions. The colour scale on the right-hand side of each panel indicates the strength of positive (green) and inverse (red) correlations
