# Supplementary figures and images for: Network analysis of nitrate-sensitive oral microbiome reveals interactions with cognitive function and cardiovascular health across dietary interventions
Source: Redox Biol. 2021 Mar 5;41:101933. doi: 10.1016/j.redox.2021.101933 (PMC7970425; doi:10.1016/j.redox.2021.101933)

A

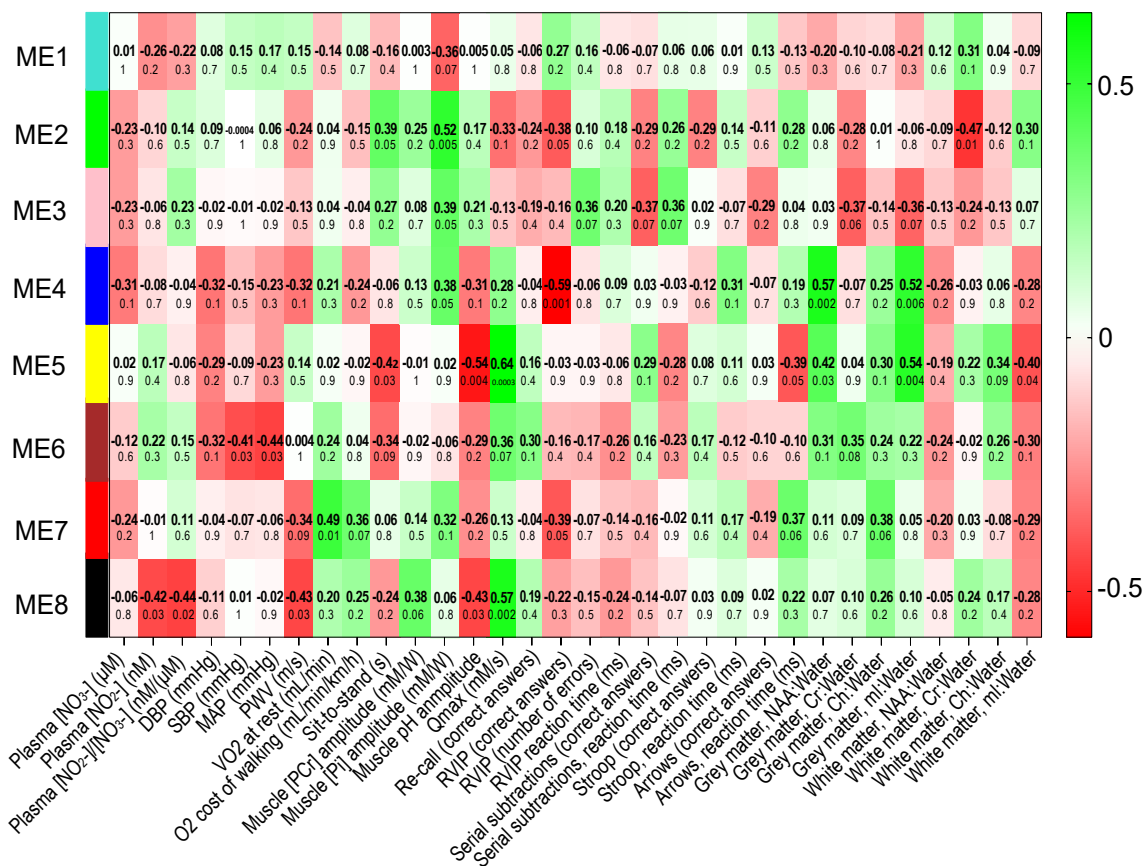

B

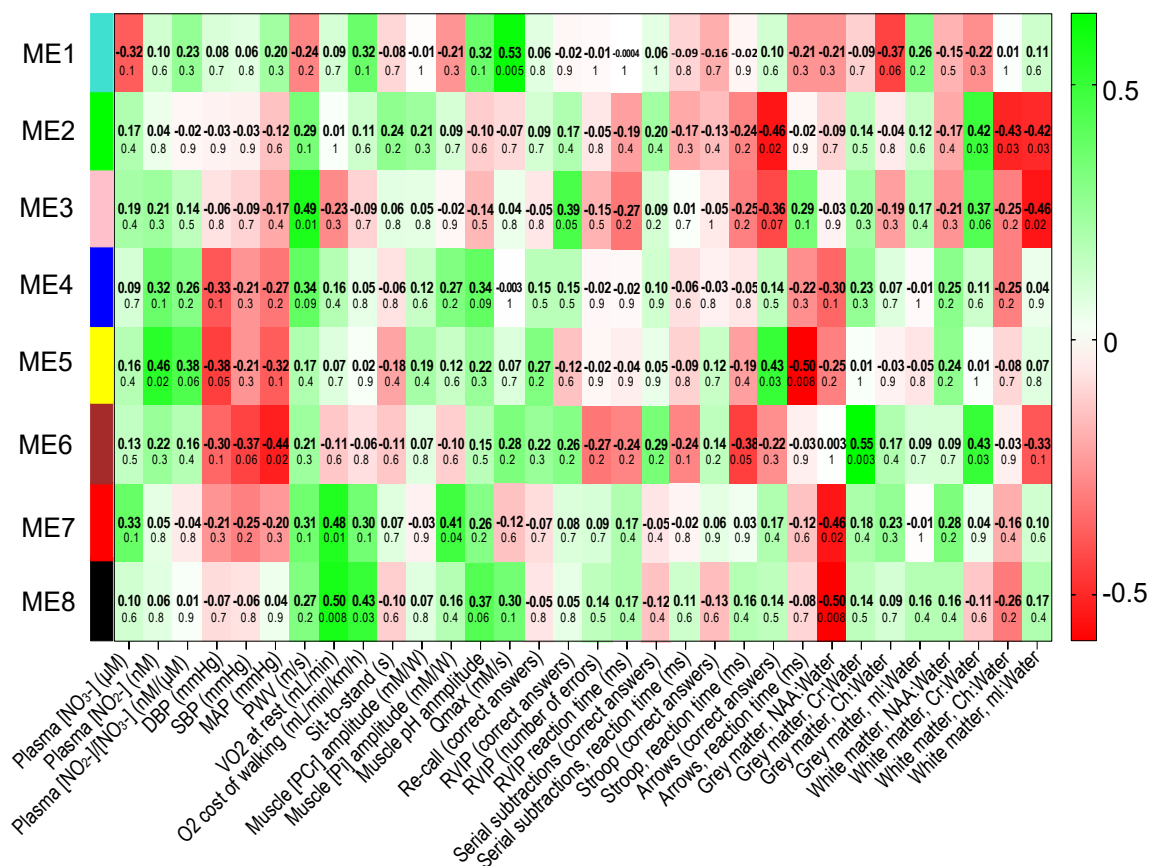

Supplement: Multimedia component 3 [file mmc3.pdf]
